# Bacterial Pericarditis Caused by Campylobacter fetus subsp. fetus After Mutton Consumption

**DOI:** 10.7759/cureus.33213

**Published:** 2023-01-01

**Authors:** Takaaki Shigematsu, Yasushi Shibue

**Affiliations:** 1 Infectious Disease, Yokohama City Minato Red Cross Hospital, Yokohama, JPN; 2 Diabetes and Endocrinology, The Fraternity Memorial Hospital, Tokyo, JPN

**Keywords:** zoonoses, blood stream infection, bacteremia, foodborne illness, undercooked meat, raw meat, pericarditis, acute pericarditis, campylobacteriosis, campylobacter fetus

## Abstract

*Campylobacter fetus* subsp. *fetus* causes systemic diseases including bacteremia and meningitis. However, it rarely causes bacterial pericarditis. We present a rare case of bacterial pericarditis caused by *Campylobacter fetus *subsp. *fetus*.

A man in his 60s presented with a fever and dyspnea. Electrocardiography revealed ST segment elevation in all leads except augmented vector right (aVR), and contrast-enhanced computed tomography of the chest revealed a large pericardial effusion. *Campylobacter fetus* subsp. *fetus*, appearing as curved, gull-wing-shaped gram-negative rods on microscopy, was identified on blood culture. The patient was diagnosed with acute pericarditis caused by *Campylobacter fetus* subsp. *fetus*. Further, history-taking revealed that he had consumed undercooked mutton before the onset of his illness. He recovered after treatment with antibiotics (ceftriaxone, ampicillin, and amoxicillin) for four weeks. With a blood culture revealing gull-wing shaped gram-negative rods, and the patient’s history including potential contact with animals or the consumption of raw or undercooked meat, *Campylobacter fetus* subsp. *fetus* infection should be suspected.

## Introduction

*Campylobacter fetus* subsp. *fetus *is a gram-negative rod that is primarily hosted by cattle and sheep. It is covered with a layer of surface array protein (S protein) called crystalline surface layer (S-layer) [1]. S-layer plays the role of serum resistant and anti-phagocytic effect to protect *Campylobacter fetus* subsp. *fetus* from immune function. Therefore, it is also well known for causing bacteremia and meningitis [2]. On the other hand, reports of pericarditis with *Campylobacter fetus* subsp. *fetus* infections are rare, with only 11 cases reported thus far. In addition to the present case, only one other case included the consumption of raw or undercooked meat in the patient’s history.

## Case presentation

A man in his 60s presented with a two-week history of fever and arthralgia and a three-day history of left anterior chest pain. He previously visited a clinic and was treated with azithromycin for three days; however, his symptoms did not improve. He developed dyspnea on exertion, which led him to seek care at our hospital. He had a history of a postoperative anal fistula, atrial fibrillation, type 2 diabetes mellitus, dyslipidemia, and hyperuricemia. He smoked 40 cigarettes per day for 34 years, until he was 54 years old. He drank shochu, a type of Japanese distilled spirit, daily.

On admission to the emergency department, he was alert, orientated, and fully conscious. He had an axillary temperature of 36.3°C, blood pressure of 129/75 mmHg, pulse rate of 92 beats/min with an irregular rhythm, respiratory rate of 21 breaths/min, and oxygen saturation of 95% while receiving oxygen (2 L/min) via a nasal cannula. He reported left anterior chest pain, which was not exacerbated by compression. No evidence of petechial hemorrhages, a pleural friction rub, or edema was observed. His lungs were clear on auscultation, and no abnormalities were noted on abdominal examination.

Blood tests revealed neutrophilic leukocytosis, thrombocytosis, liver dysfunction, and elevated levels of C-reactive protein and brain natriuretic peptide. However, his cardiac enzyme levels were normal. His renal and thyroid function test results were almost within normal limits (blood urea nitrogen was above the normal range). His diabetes was well-controlled. Autoantibodies and virus antibody titers test results were unremarkable (Table 1).

**Table 1:** Summary of the laboratory studies during the first hospital admission

Measure	Reference	Result
White blood cell (10^3/μL)	3.9-9.8	12.6
Neutrophils (%)	40-74	80
Hemoglobin (g/dL)	13.5-17.6	16.2
Platelets (10^3/μL)	13.1-36.2	437
Aspartate transaminase (U/L)	10-40	91
Alanine transaminase (U/L)	5-40	146
Alkaline phosphatase (U/L)	115-359	857
Blood urea nitrogen (mg/dL)	8.0-22.0	24.5
Creatinine (mg/dL)	0.17-1.00	1.00
C-reactive protein (mg/dL)	≦0.14	33.2
Brain natriuretic peptide (pg/mL)	≦18.4	241.3
Creatine kinase (U/L)	62-287	25
Creatine kinase-MB (U/L)	≦25	4
Troponin I	26.2	0.004ng/mL
Thyroid stimulating hormone (μU/mL)	0.5-5.00	1.772
Triiodothyronine (pg/mL)	2.3-4.3	1.76
Thyroxine (ng/dL)	0.9-1.7	1.22
Hemoglobin A1c (%)	4.6-6.2	6.4
Blood glucose level (mg/dL)	70-109	143
Anit-SS-A antibody (U/mL)	<10	<0.5
Anti-SS-B antibody (U/mL)	<10	<0.5
Anti-SM antibody (U/mL)	<10	<0.5
Proteinase3 antineutrophil cytoplasmic antibody (U/mL)	<3.5	<0.5
Myeloperoxidase antineutrophil cytoplasmic antibody (U/mL)	<3.5	<0.5
Rheumatoid factor (IU/mL)	15	12
Anti-Cyclic citrullinated Peptide (U/mL)	<4.5	<0.5
Anti-nuclear antibody (fold)	<40	<40
Parainfluenza type 1 antibody (fold)	<10	<10
Parainfluenza type 2 antibody (fold)	<10	<10
Parainfluenza type 3 antibody (fold)	<10	20
Echovirus type 1 antibody (fold)	<4	<4
Echovirus type 6 antibody (fold)	<4	32
Echovirus type 9 antibody (fold)	<4	8
Echovirus type 19 antibody (fold)	<4	<4
Coxsackievirus B1 antibody (fold)	<4	<4
Coxsackievirus B2 antibody (fold)	<4	<4
Coxsackievirus B3 antibody (fold)	<4	<4
Coxsackievirus B4 antibody (fold)	<4	<4
Coxsackievirus B5 antibody (fold)	<4	<4
Mumps virus antibody (fold)	<4	<4
Herpes simplex virus antibody	<4	<4
Cytomegalovirus IgM (AU/mL)	<0.85	0.25
Cytomegalovirus IgG (AU/mL)	<6	18
Epstein-Barr virus (EBV) viral capsid antigen (VCA) IgM (fold)	<10	0
EBV VCA IgG (fold)	<10	2.2
EBV nuclear antigen antibody (fold)	<10	40

**Figure 1:**
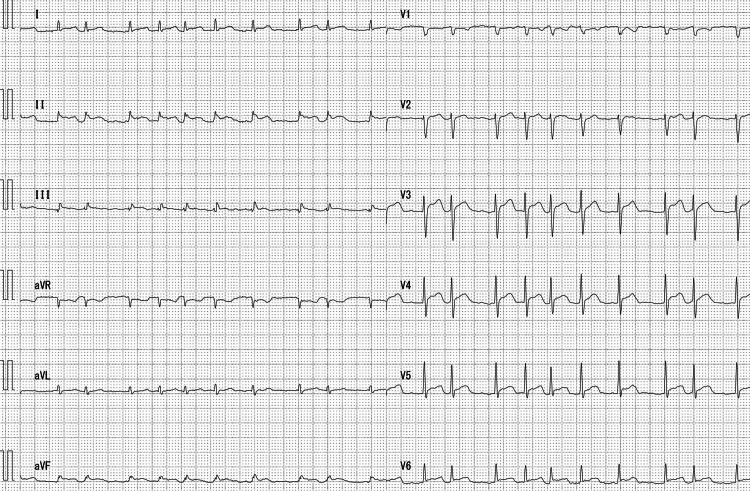
Electrocardiogram on admission Electrocardiogram performed on day 1 of hospital admission. The electrocardiogram shows ST segment elevation in all leads except aVR (fourth row). aVR: augmented vector right

**Figure 2:**
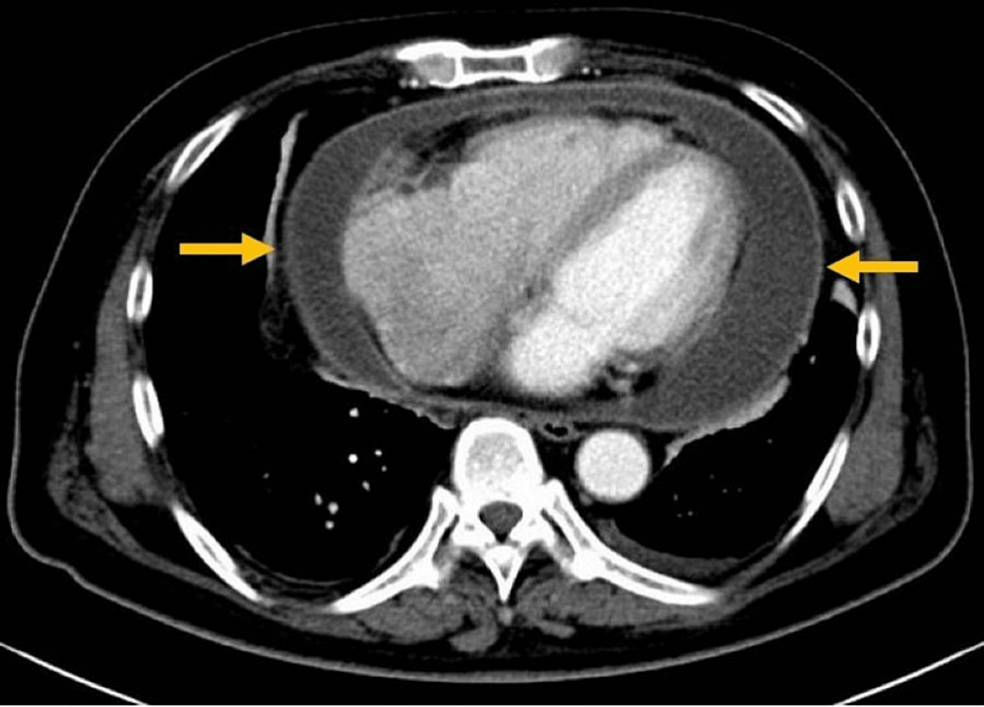
CT scan of chest on admission showed pericardial effusion CT: computed tomography

Electrocardiography showed atrial fibrillation and ST-segment elevation in all leads except augmented vector right (aVR) (Figure 1). Chest radiography revealed cardiomegaly (cardiothoracic ratio: 75.7%), with no lung infiltration or masses identified in the lung fields. Transthoracic echocardiography revealed a massive pericardial effusion with a depth of approximately 1-3 cm; however, the patient’s cardiac function was preserved (ejection fraction approximately 60%, with no chamber collapse). Echocardiography did not show any vegetation on the heart valves (Video 1). Contrast-enhanced computed tomography of the chest revealed pericardial thickening and effusion, pulmonary interstitial thickening (due to blood congestion in the lungs), and small bilateral pleural effusions (Figure 2). Abdominal computed tomography showed a hepatic hemangioma but no other abnormalities.

**Video 1:** Transthoracic echocardiography before treatment

Based on these results, the patient was admitted to the department of cardiology with a diagnosis of acute pericarditis for further investigation and treatment. After obtaining samples for various cultures, he was started on oral loxoprofen (60 mg, three times a day). On the third day of hospitalization, gram-negative rods with spiral, curved gull-wing shapes were found in one of the two sets of blood cultures submitted on admission, which were subsequently identified as *Campylobacter fetus *subsp. *fetus*. We subsequently asked the patient about possible sources of exposure and he disclosed that he had been in the habit of eating raw meat on a regular basis and that he had eaten some undercooked mutton approximately one week before the onset of symptoms.

Differential diagnosis

We hypothesized that the cause of acute pericarditis could be viral, autoimmune, or bacterial. Tests for autoimmune diseases were all negative. Regarding a viral cause, only parainfluenza type 3 antibodies increased (4-fold; from 20-fold to 80-fold) in paired sera. However, blood cultures taken more than 24 hours apart showed *Campylobacter fetus* subsp. *fetus* in both cases, leading to the diagnosis of bacterial pericarditis.

Treatment

Treatment with intravenous ceftriaxone 2 g every 24 hours was started on the day the blood culture results turned positive. When a sensitivity test revealed that the organism was susceptible to all antimicrobial agents tested, the antibiotic was switched to intravenous ampicillin (2 g every 6 hours) based on the antibiotic susceptibility results. After the start of antimicrobial treatment, the patient's fever gradually resolved, his white blood cell count and blood C-reactive protein level decreased, and the volume of pericardial fluid also decreased. After three weeks, intravenous ampicillin treatment ceased, and the patient was switched to oral amoxicillin (1500 mg/day) for one week. After responding favorably to treatment, the patient was discharged on the 28th day.

Outcome and follow-up

At his first follow-up visit four weeks after discharge, he had no symptoms of dyspnea, arthralgia, or chest pain, and transthoracic echocardiography showed decreased pericardial effusion (from 29 mm to 5 mm behind the left ventricle) (Video 2).

**Video 2:** Transthoracic echocardiography after treatment

## Discussion

Acute pericarditis has been attributed to viral, bacterial, and autoimmune diseases. While the majority of cases in developed countries are idiopathic, most of them are considered to be of viral origin [3]. In this case, a 4-fold increase in the antibody titer of parainfluenza virus type 3 (from 20-fold to 80-fold) was observed in paired sera. However, *Campylobacter fetus *subsp.* fetus* was detected in a blood culture collected on the day of admission and in a blood culture collected more than 24 hours later (before the initiation of antimicrobial agents), indicating persistent bacteremia with *Campylobacter fetus *subsp. *fetus. *Therefore, the patient was diagnosed with bacterial pericarditis caused by *Campylobacter fetus *subsp. *fetus *and treated accordingly.

Bacteria in the Campylobacter genus are curved, gull-wing-shaped gram-negative rods. *Campylobacter jejuni *and *Campylobacter fetus* subsp. *fetus* are the most common human pathogens in this genus. *Campylobacter jejuni *and *Campylobacter fetus* subsp. *fetus *have been studied in veterinary medicine as causative agents of abortion and enteritis in cattle, pigs, and sheep, and enteritis in chickens [4]. Human infection was first reported in 1913 when *Campylobacter fetus* subsp. *fetus* was detected in a uterine discharge [5].

*Campylobacter jejuni *and* Campylobacter fetus *subsp. *fetus *are both members of the Campylobacter genus; however, there are some differences between them (Table 2). For example, the hosts of *Campylobacter jejuni *are poultry, whereas the hosts of *Campylobacter fetus* subsp. *fetus *are cattle, sheep, and reptiles. Moreover, *Campylobacter jejuni* infection in humans often manifests with symptoms of acute gastroenteritis, whereas *Campylobacter fetus *subsp. *fetus* is characterized by systemic diseases such as bacteremia and meningitis [6]. The case presented is consistent with previous reports because *Campylobacter fetus* subsp. *fetus *was detected in blood culture, the patient had extraintestinal symptoms, and he reported exposure to undercooked mutton.

**Table 2:** Comparison between the characteristics of epidemiologic, laboratory, and clinical characteristics of Campylobacter jejuni and Campylobacter fetus subsp. fetus

Feature	Campylobacter jejuni	Campylobacter fetus subsp. fetus
Epidemiologic characteristics		
Major reservoir	Poultry	Cattle and sheep, reptiles
Affected hosts	Normal hosts; all ages affected; cases often occur in clusters	Opportunistic agent in debilitated hosts; clustering rare; healthy hosts may be affected
Laboratory characteristics		
Range of growth temperatures	32-42°C	25-37°C (Occasionally grows up to 42°C)
Usual source of isolation	Feces	Blood
Clinical characteristics		
As a cause for diarrheal illness	Common	Uncommon
Clinical manifestations	Acute gastroenteritis, colitis	Systemic illness with bacteremia, meningitis, vascular infections, abscesses; gastroenteritis
Outcome of infection	Usually self-limited	Potentially fatal in debilitated hosts

*Campylobacter jejuni *causes intestinal infections, whereas *Campylobacter fetus *subsp. *fetus* causes extraintestinal infections. The surface array protein (S protein), which covers the surface layer of *Campylobacter fetus *subsp. *fetus*, is believed to be involved in this process. The S protein covers the surface layer of the bacterial body, enabling it to resist phagocytosis by leukocytes in the same way that bacteria with capsular membranes do. In addition, as the lipopolysaccharide to which the complement binds is covered with S protein, complement components are less likely to bind to the bacterial body. Mouse experiments have demonstrated that bacteria covered with S proteins are about 10 times more likely to cause bacteremia than those lacking S proteins [7]. Furthermore, by altering the antigenicity and morphology of S proteins, they can escape from the host humoral immune response [8].

Although* Campylobacter fetus *subsp. *fetus* is known to cause extraintestinal infections, such as meningitis and bacteremia, there have been few reports of pericarditis. A literature search revealed only 11 previously reported cases [9-19]. These are presented in chronological order in Table 3. In four cases, the patients had a clear history of animal contact as a possible source of infection, and one previous case was associated with the consumption of raw beef [19]. In six cases, the patients had factors associated with immunodeficiencies, such as diabetes, hemodialysis, or malignancy [12,15-19]. The patient in our case study had a history of diabetes mellitus, which was consistent with previous evidence that* Campylobacter fetus* subsp. *fetus *causes extraintestinal infections in immunocompromised patients.

**Table 3:** Summary of 12 cases of pericarditis caused by Campylobacter fetus subsp. fetus

Case	Age (years), sex	Positive material	Underlying disease	Animal exposure	Prodrome (duration)	Antimicrobial treatment (duration)	Outcome	Reference
1	68 M	Blood	Gallstone	Farmer	Intermittent fever, chills (1 month)	PC, CTET (2 months)	Recovered	[9]
2	48 F	Blood, PE	Possible rheumatic fever	Living on a cattle farm, caring for sick calves	Chills, fever, malaise, weakness (3 weeks)	Procaine PC, PCG (5 weeks), CP (4 weeks)	Hemiparesis	[10]
3	38 F	PE	None	Living in a rural area	Dyspnea (2 weeks), bilateral lower chest pain, dry cough, nausea	SM, INH, PAS, RFP, CET, PC, GM	Death	[11]
4	60 F	Blood, PE	Lymphoma	ND	Abdominal pain, diarrhea, vomiting	ND	Death	[12]
5	57 F	PE	Hypothyroidism	Dog at home	Cough, fever, anorexia, weight loss (2 weeks)	EM, ABPC (4 weeks)	Recovered	[13]
6	36 M	Blood	Polycystic kidney, hypertension	ND	Fever, dry cough	ABPC, EM, DOXY (ND)	Recovered	[14]
7	61 F	Blood	Hypertension, T2DM	None	Nausea, myalgia, headache, chest pain, sweats, dyspnea (1 week)	EM, GM (4 weeks)	Recovered	[15]
8	24 F	Blood, PE	β-thalassemia, splenectomy, chronic transfusion and deferoxamine treatment	None	Chill, fever, odynophagia, chest pain (15 days)	PC, GM (30 days)	Recovered	[16]
9	14 F	PE	β-thalassemia, splenectomy	None	Fever, chill, vomiting, diarrhea (5 days)	CTRX, CXM, VCM, CAM, ABPC (3 weeks), AMPC (3 weeks)	Recovered	[17]
10	70 M	Blood, PE	DM, CAD	None	Diarrhea (2-3 days before onset)	IPM, CS (ND)	Recovered	[18]
11	62 M	PE	Hemodialysis, graft on abdominal aorta, renal cancer, cecal cancer	Ingestion of raw bovine liver	Fever (5 days)	VCM, CPFX (4 weeks)	Recovered	[19]
12	68 M	Blood	anal fistula, atrial fibrillation, T2DM, dyslipidemia, and hyperuricemia	Ingestion of raw mutton meat	Fever, arthralgia (12 days), chest pain, dyspnea (3 days)	CTRX (8 days), ABPC (13 days), AMPC (7 days)	Recovered	Present case

Nine of the 11 historical patients recovered [9,13-19], two died [11,12], and one had residual hemiplegia [10]. In the cases that were successfully treated, ampicillin, gentamicin, and carbapenems were used as antimicrobial treatments. Most patients were treated with antimicrobials for more than four weeks. As the *Campylobacter fetus* subsp. *fetus *in this case was susceptible to all the antibiotics tested, we administered ceftriaxone, ampicillin, and oral amoxicillin sequentially for a total of four weeks.

## Conclusions

*Campylobacter fetus* subsp.* fetus* is well-known for causing systemic infections such as bacteremia and meningitis in immunocompromised patients and it rarely causes acute pericarditis. Thus, when gull-wing-shaped gram-negative rods are detected in blood cultures, it is essential to inquire about the history of contact with animals or consumption of raw meat, if not done already, and consider the possibility of *Campylobacter fetus* subsp. *fetus* when choosing an antimicrobial agent.
